# Investigation of cyanine dyes for in vivo optical imaging of altered mitochondrial membrane potential in tumors

**DOI:** 10.1002/cam4.252

**Published:** 2014-04-16

**Authors:** Satoru Onoe, Takashi Temma, Yoichi Shimizu, Masahiro Ono, Hideo Saji

**Affiliations:** 1 Department of Patho-Functional Bioanalysis, Graduate School of Pharmaceutical Sciences, Kyoto UniversityKyoto, Japan

**Keywords:** Albumin binding, cancer diagnosis, mitochondrial membrane potential, near infrared, optical imaging

## Abstract

Mitochondrial membrane potential (Δ*ψ*_m_) alteration is an important target for cancer diagnosis. In this study, we designed a series of near-infrared fluorescent cationic cyanine dyes with varying alkyl chain lengths (IC7-1 derivatives) to provide diverse lipophilicities and serum albumin-binding rates, and we evaluated the usefulness of these derivatives for in vivo Δ*ψ*_m_ imaging. IC7-1 derivatives with side chains from methyl to hexyl (IC7-1-Me to IC7-1-He) were synthesized, and their optical properties were measured. Cellular uptake and intracellular distribution were investigated with depolarized HeLa cells from carbonyl cyanine *m*-chlorophenylhydrazone (CCCP) treatment using a spectrofluorometer and a fluorescence microscope. Serum albumin-binding rates were evaluated using albumin-binding inhibitors. In vivo optical imaging was performed with HeLa cell xenograft mice following intravenous administration of IC7-1 derivatives with or without warfarin and CCCP as in vivo blocking agents. IC7-1 derivatives showing maximum excitation and emission wavelengths at 823 nm and ∼845 nm, respectively, were synthesized. IC7-1-Me to -Bu showed fluorescence in mitochondria that decreased with CCCP treatment in a concentration-dependent manner, which showed that IC7-1-Me to -Bu successfully indicated Δ*ψ*_m_. Tumors were clearly visualized after IC7-1-Bu administration. Treatment with warfarin or CCCP significantly decreased IC7-1-Bu fluorescence in the tumor region. In summary, IC7-1-Bu exhibited fluorescence localized to mitochondria dependent on Δ*ψ*_m_, which enabled clear in vivo tumor imaging via serum albumin as a drug carrier for effective tumor targeting. Our data suggest that IC7-1-Bu is a promising NIR probe for in vivo imaging of the altered Δ*ψ*_m_ of tumor cells.

## Introduction

Cancer is the second leading cause of death worldwide, and it is thought that cancer mortality rates will continue to increase 1. It has been reported that mitochondria are an important cancer therapeutic target since they are associated with fundamental cellular functions such as energy production and regulation of the intrinsic apoptosis pathway, and as such, mitochondria have been implicated in multiple aspects of tumorigenesis and tumor progression 2–5. Since mitochondrial and nuclear DNA mutations and oxidative stress cause mitochondrial membrane potential (Δ*ψ*_m_) alteration, an important characteristic of cancer 6–8, compounds which accumulate in hyperpolarized mitochondria can be used as the core structures of tumor imaging agents and tumor targeting drugs 9–13.

Molecular imaging is an evolving field that is progressing from basic research to use in clinical diagnosis 14,15. Among several imaging methods, optical imaging can conveniently and safely offer pronounced spatial and temporal resolution. In particular, near-infrared (NIR) fluorescent probes that emit fluorescence in the NIR region (700–900 nm) are desirable for in vivo applications due to high tissue permeability 16,17. Considering the delivery and accumulation of molecular probes for imaging cancer mitochondria, molecular probes should be sufficiently lipophilic for transmittance across cellular and mitochondrial membranes as well as cationic to stay in the negatively charged environment of the cancer mitochondrial matrix 2,9. While fluorescent delocalized lipophilic cations (DLCs) have been developed for this purpose, they fluoresce in the visible region, which is useful for bioassay or fluorescence microscopy 18,19; however, NIR-DLC probes have not yet been reported. Thus, the goal of this study was to develop a NIR-DLC probe for in vivo optical imaging of tumors with altered Δ*ψ*_m_ based on the structure of the NIR fluorescent cyanine dye IC7-1 (*λ*ex = 830 nm, *λ*em = 858 nm) that we had previously developed 16.

In this study, we synthesized IC7-1 derivatives possessing various alkyl side chain lengths for lipophilicity optimization as mitochondrial imaging probes (Scheme5), evaluated their sensitivity to changes of membrane potential in cellular uptake and NIR fluorescence microscopy studies, and investigated their usefulness as tumor imaging agents using tumor-bearing mice and an in vivo imaging modality, Clairvivo OPT, which is designed for NIR imaging of small animals 16. We found that the length of the alkyl chain of the IC7-1 derivative greatly affected not only the membrane potential sensitivity but also the probe biodistribution in tumor-bearing mice.

**Figure 5:**
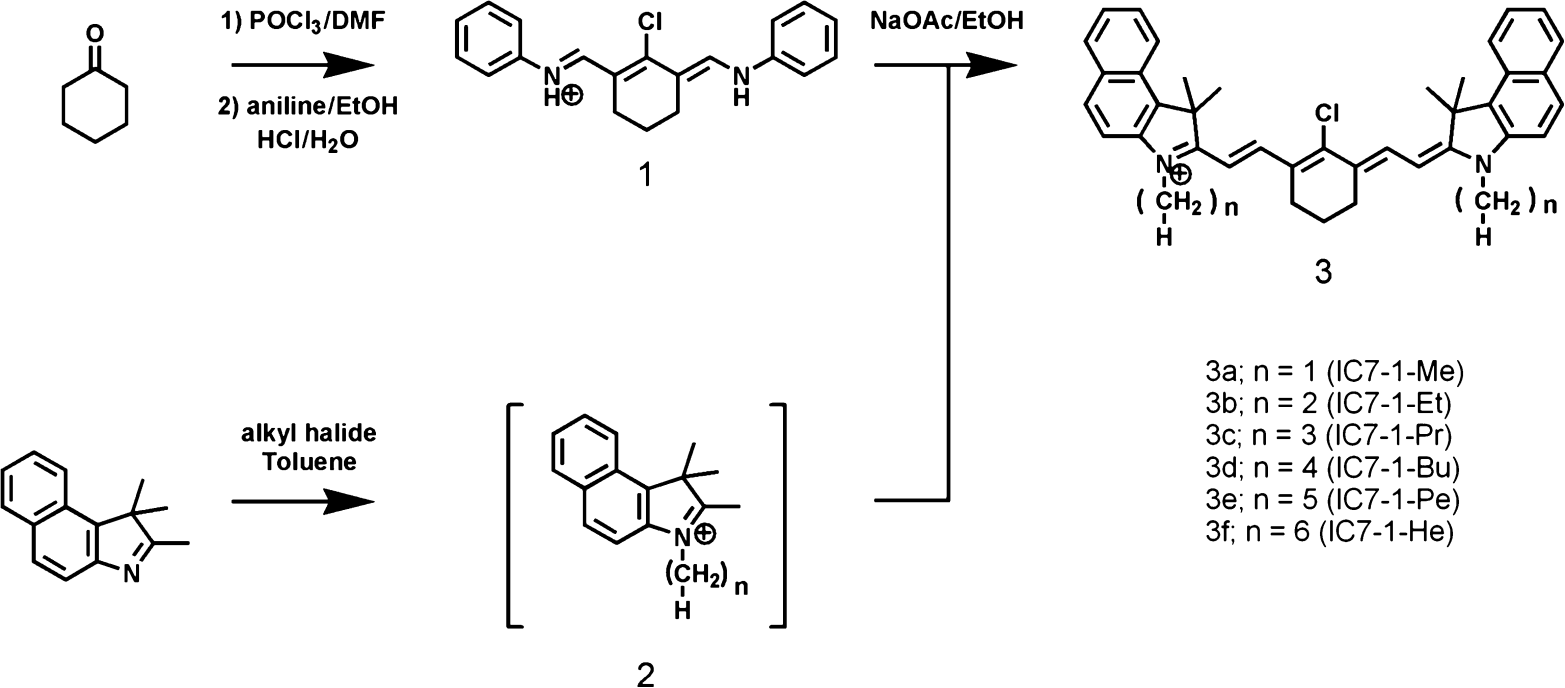
Scheme Synthesis of IC7-1 derivatives.

## Material and Methods

### Materials

All reagents were purchased from Wako Pure Chemical Industries, Ltd. (Osaka, Japan), Tokyo Chemical Industry Co, Ltd. (Tokyo, Japan), or Nacalai Tesque Inc. (Kyoto, Japan) and were used without further purification. For cell experiments, Dulbecco's modified Eagle's medium (DMEM), fetal bovine serum (FBS), and nonyl acridine orange (NAO) were purchased from Nissui Pharmaceutial Co., Ltd. (Tokyo, Japan), Nichirei Co. (Tokyo, Japan), and Biotium, Inc. (Hayward, CA), respectively. D10001 was purchased from Research Diets, Inc. (New Brunswick, NJ)

### Instruments

^1^H-NMR spectra were recorded on a JEOL ECP-300 (JEOL Ltd., Tokyo, Japan). Mass spectra were acquired on a SHIMADZU LC-MS2010 EV (SHIMADZU Co., Kyoto, Japan). UV-vis spectra were measured using a UV-1800 (SHIMADZU Co.). Cells were imaged with a fluorescence microscope (IX81N-ZDC-IMAGE; Olympus Co., Kyoto, Japan) equipped with a CCD camera for NIR light (1024B_eXcelon; Princeton Instruments, Trenton, NJ) using U-MNIGA3 filter sets for NAO and a custom filter set for IC7-1 (MX0820 (Asahi Spectra Co. Ltd., Tokyo, Japan) for excitation, and MX0860 (Asahi Spectra Co. Ltd.) for emission, and 845DRSP (Omega Optical Inc., Brattleboro, VT) for the dichroic mirror under a 100× oil-immersion objective lens. Fluorescence spectroscopy was performed with a Fluorolog-3 equipped with a NIR-sensitive photomultiplier detection system (∼1200 nm) (Horiba Jobin Yvon Inc., Kyoto, Japan), using a slit width of 10 nm for excitation measurements and 5 nm for emission measurements.

### Synthesis

*N*-[5-anilino-3-chloro-2,4-(propane-1,3-diyl)-2,4- pentadiene-1-ylidene]anilinium chloride (1) was prepared as reported previously (10.5 g, 56.8%) 16 (Scheme5).

#### General procedure for the synthesis of 3-substituted 2-(2-{3-[2-(3-substituted -1,1-dimethyl-1,3-dihydro-benz[e]indol-2-ylidene)-ethylidene]-2-chloro-cyclohex-1- enyl}-vinyl)-1,1-dimethyl-1H-benz[e]indolium 3a–3f

1,1,2-Trimethyl-1*H*-benz[*e*]indole (2.16 g, 10.3 mmol) was added to the alkyl halide (30.9 mmol) dissolved in toluene (3.6 mL), and the mixture was refluxed for 12 h. The resulting precipitate was washed with ether and hexane, and then dried in vacuo. The product was used for the next reaction without further purification. The resulting compound (1000 mg, 2.5 eq) was mixed with compound 1 (384–477 mg, 1 eq) and anhydrous sodium acetate (88–95 mg, 2.5 eq) in EtOH (15 mL), and the mixture was refluxed for 3 h. After completion of the reaction, the mixture was cooled to room temperature, and the solvent was evaporated. The reaction mixture was partitioned between chloroform and water, and the organic layer was separated, washed with brine, dried over anhydrous magnesium sulfate (MgSO_4_), and concentrated under vacuum. The residue was purified by column chromatography to obtain compound 3a–3f.

#### 3-Methyl-2-(2-{3-[2-(3-methyl-1,1-dimethyl-1,3-dihydro-benz[e]indol-2-ylidene)-ethylidene]-2-chloro-cyclohex-1-enyl}-vinyl)-1,1-dimethyl-1H-benz[e]indolium (3a)

Compound (3a) was obtained from iodomethane as a dark-green solid (694.6 mg, 66.2%). ^1^H NMR (CDCl_3_) *δ*8.46–8.42 (m, 2H), 8.14–8.11 (m, 2H), 7.97–7.93 (m, 4H), 7.63–7.59 (m, 2H), 7.52–7.46 (m, 4H), 6.34 (d, 2H), 3.91 (s, 6H), 2.80 (t, 4H), 2.03 (s, 12H), 1.64 (m, 2H): MS (ESI, pos) *m/z* calcd. for C_40_H_40_ClN_2_ (M+): 583; *m/z* found: 583.

#### 3-Ethyl-2-(2-{3-[2-(3-ethyl-1,1-dimethyl-1,3-dihydro-benz[e]indol-2-ylidene)-ethylidene]-2-chloro-cyclohex-1-enyl}-vinyl)-1,1-dimethyl-1H-benz[e]indolium (3b)

Compound (3b) was obtained from iodoethane as a dark-green solid (454 mg, 67.8%). ^1^H NMR (CDCl_3_) *δ*8.47–8.44 (m, 2H), 8.14–8.12 (m, 2H), 7.97–7.94 (m, 4H), 7.63–7.60 (m, 2H), 7.52–7.43 (m, 4H), 6.32 (d, 2H), 4.44–4.38 (m, 4H), 2.81 (t, 4H), 2.03 (s, 12H), 1.55–1.51 (m, 8H): MS (ESI, pos) *m/z* calcd. for C_42_H_44_ClN_2_ (M+): 611; *m/z* found: 611.

#### 3-Propyl-2-(2-{3-[2-(3-propyl-1,1-dimethyl-1,3-dihydro-benz[e]indol-2-ylidene)-ethylidene]-2-chloro-cyclohex-1-enyl}-vinyl)-1,1-dimethyl-1H-benz[e]indolium (3c)

Compound (3c) was obtained from 1-iodopropane as a dark-green solid (516.4 mg, 58.0%). ^1^H NMR (CDCl_3_) *δ*8.46–8.43 (m, 2H), 8.14–8.12 (m, 2H), 7.96–7.94 (m, 4H), 7.63–7.60 (m, 2H), 7.52–7.43 (m, 4H), 6.30 (d, 2H), 4.35–4.31 (m, 4H), 2.80 (t, 4H), 2.03 (s, 12H), 2.03–1.96 (m, 4H), 1.65–1.55 (m, 2H), 1.11 (t, 6H): MS (ESI, pos) *m/z* calcd. for C_44_H_48_ClN_2_ (M+): 639; *m/z* found: 639.

#### 3-Butyl-2-(2-{3-[2-(3-butyl-1,1-dimethyl-1,3-dihydro-benz[e]indol-2-ylidene)-ethylidene]-2-chloro-cyclohex-1-enyl}-vinyl)-1,1-dimethyl-1H-benz[e]indolium (3d)

Compound (3d) was obtained from 1-bromobutane as a dark-green solid (738.6 mg, 80.6%). ^1^H NMR (CDCl_3_) *δ*8.46–8.43 (m, 2H), 8.14–8.12 (m, 2H), 7.96–7.94 (m, 4H), 7.64–7.60 (m, 2H), 7.50–7.44 (m, 4H), 6.34 (d, 2H), 4.40–4.36 (m, 4H), 2.79 (t, 4H), 2.03 (s, 12H), 1.94–1.86 (m, 4H), 1.71 (m, 2H), 1.59–1.49 (m, 4H), 1.11 (t, 6H): MS (ESI, pos) *m/z* calcd. for C_46_H_52_ClN_2_ (M+): 667, *m/z* found: 667.

#### 3-Pentyl-2-(2-{3-[2-(3-pentyl-1,1-dimethyl-1,3-dihydro-benz[e]indol-2-ylidene)-ethylidene]-2-chloro-cyclohex-1-enyl}-vinyl)-1,1-dimethyl-1H-benz[e]indolium (3e)

Compound (3e) was obtained from 1-bromopentane as a dark-green solid. (610.9 mg, 74.3%). ^1^H NMR (CDCl_3_) *δ*8.46–8.43 (m, 2H), 8.14–8.12 (m, 2H), 7.96–7.94 (m, 4H), 7.64–7.60 (m, 2H), 7.52–7.43 (m, 4H), 6.34 (d, 2H), 4.38-4.34 (m, 4H), 2.79 (t, 4H), 2.03 (s, 12H), 1.97–1.86 (m, 4H), 1.70–1.65 (m, 2H), 1.52-1.38 (m, 8H), 0.93 (t, 6H): MS (ESI, pos) *m/z* calcd. for C_48_H_56_ClN_2_ (M+): 695; *m/z* found: 695.

#### 3-Hexyl-2-(2-{3-[2-(3-hexyl-1,1-dimethyl-1,3-dihydro-benz[e]indol-2-ylidene)-ethylidene]-2-chloro-cyclohex-1-enyl}-vinyl)-1,1-dimethyl-1H-benz[e]indolium (3f)

Compound (3f) was obtained from 1-bromohexane as a dark-green solid. (682.2 mg, 73.3%). ^1^H NMR (CDCl_3_) *δ*8.46–8.43 (m, 2H), 8.14–8.12 (m, 2H), 7.96–7.94 (m, 4H), 7.64–7.60 (m, 2H), 7.52–7.43 (m, 4H), 6.34 (d, 2H), 4.38–4.34 (m, 4H), 2.79 (t, 4H), 2.03 (s, 12H), 1.94–1.86 (m, 4H), 1.69 (m, 2H), 1.52–1.40 (m, 12H), 0.93 (t, 6H): MS (ESI, pos) *m/z* calcd. for C_50_H_60_ClN_2_ (M+): 723; *m/z* found: 723.

### Photophysical properties

IC7-1 derivatives were dissolved in chloroform or 5% FBS to give a concentration of 1 *μ*mol/L for fluorescence spectroscopy analysis. Excitation spectra were measured following the emission at 850 nm, and emission spectra were measured following excitation at 823 nm. Quantum yields were determined using a Fluorolog-3 spectrofluorometer equipped with an integrating sphere.

### Cell culture

The human cervix adenocarcinoma HeLa cell line was obtained from American Type Culture Collection and was authenticated with the Promega PowerPlex® 16 STR system (Madison, WI) in October 2012. HeLa cells were grown in DMEM supplemented with 10% heat-inactivated FBS and 1% penicillin and streptomycin. Cells were maintained in a humidified atmosphere containing 5% CO_2_ at 37°C.

### Cellular localization study

HeLa cells (3 × 10^5^ cells) were cultured in a poly-l-lysine coated glass bottom dish for 24 h at 37°C before use. Cells were washed twice with phosphate-buffered saline (PBS) and then incubated with the IC7-1 derivative dissolved in dimethyl sulfoxide (DMSO) (1 *μ*mol/L final concentration) in DMEM supplemented with 5% FBS and 1% penicillin and streptomycin for 60 min. To determine the dye localization, mitochondria were labeled with NAO (100 nmol/L final concentration) for 20 min before completion of the incubation with IC7-1 derivative. Next, the cells were washed twice each with DMEM and PBS. Cells were viewed with a fluorescence microscope under a 100× oil-immersion objective lens. MetaMorph® software (Molecular Devices, LLC, Sunnyvale, CA) was used for imaging analysis.

### Cellular uptake study

A cellular uptake study was conducted as reported previously 8. Briefly, the buffer solution (pH 7.4) for the cellular uptake study was composed of NaCl (145 mmol/:), KCl (5.4 mmol/L), CaCl_2_ (1.2 mmol/L), MgSO_4_ (0.8 mmol/L), NaH_2_PO_4_ (0.8 mmol/L), dextrose (5.6 mmol/L), and 4-(2-hydroxyethyl)-1-piperazineethanesulfonic acid) (5 mmol/L). HeLa cells (1 × 10^6^ cells/mL buffer solution) were incubated with carbonyl cyanine *m*-chlorophenylhydrazone (CCCP; 0, 0.1, 0.25, 0.5, 0.75, 1, 2.5, 5, 10, and 25 *μ*mol/L) for 5 min at 37°C in 1.5 mL microtubes, followed by the addition of the IC7-1 derivative (1 *μ*mol/L final concentration). After a 60-min incubation with the IC7-1 derivative, the cells were washed twice and resuspended with buffer solution. The fluorescence intensity was measured following the excitation at 823 nm and the emission from 840 to 900 nm, and the normalized intensity (%) was calculated as a percentage of fluorescence intensity obtained with CCCP untreated (0 *μ*mol/L) cells.

### Protein-binding assay

A protein-binding assay was conducted as reported previously 20. Briefly, warfarin, ibuprofen, digoxin, and quinidine were used as competitive inhibitors of albumin-binding sites I, II, III, and *α*_1_-glycolipoprotein, respectively. The inhibitors (0 and 189 *μ*mol/L) were incubated for 30 min in PBS (pH 7.4) containing 5% DMSO and 5% FBS at room temperature. Immediately after the addition of the IC7-1 derivative (1 *μ*mol/L final concentration), the fluorescence intensity was measured following the excitation at 823 nm and the emission from 840 to 900 nm. The normalized intensity (%) was calculated as a percentage of fluorescence intensity obtained from an inhibitor untreated sample.

### In vivo imaging study

Animal experiments were conducted in accordance with institutional guidelines and were approved by the Kyoto University Animal Care Committee. Female nude mice (BALB/c *nu*/*nu* 4-weeks old), supplied by Japan SLC, Inc. (Shizuoka, Japan), were housed under a 12 h light/12 h dark cycle and were given free access to food (D10001) and water. HeLa cells (2 × 10^6^ cells in 100 *μ*L of PBS) were subcutaneously inoculated into the right hind legs of mice. Fourteen days after implantation, mice were used for the imaging study.

Twenty-four, 48, and 72 h after intravenous administration of the IC7-1 derivative (10 nmol, 100 *μ*L), tumor-bearing mice under anesthesia with 2.5% isoflurane gas in oxygen flow (1.5 L/min) were imaged by Clairvivo® OPT (SHIMADZU Co.) with a 785 nm single laser for excitation and a 845/55 nm band path filter for emission. Clairvivo® OPT measurement and display software ver. 2.6.0.0. (SHIMADZU Co.) was used for imaging analysis.

An in vivo blocking study using a protein-binding inhibitor was conducted as reported previously 21. Briefly, IC7-1-Bu (10 nmol, 100 *μ*L) and warfarin (5.5 *μ*mol, 100 *μ*L) were simultaneously administered to mice via the tail vein, and the mice were imaged using the same method as described above.

In the in vivo blocking study using an uncoupler, IC7-1-Bu (10 nmol, 100 *μ*L) was administered to tumor-bearing mice via the tail vein immediately after intratumoral injection of CCCP (2.5 mL/kg, 0.25 mg/kg). Mice were imaged 1 and 3 h after administration of the imaging agent using the same method as described above.

### Statistical analysis

In the cellular uptake study, data are expressed as means ± SEM. Otherwise, data are expressed as means ± SD. Data were analyzed with one-way factorial ANOVA followed by the Tukey test, and values with *P* < 0.05 were considered significant.

## Results

### Synthesis of IC7-1 derivatives

IC7-1 derivatives were synthesized from cyclohexanone and 1, 1, 2-trimethyl-1*H*-benz[*e*]indole following a similar synthetic protocol for IC7-1 as shown in Scheme5. Overall yields ranged from 31% to 44%.

### Photophysical properties of IC7-1 derivatives

The photophysical properties of IC7-1 derivatives in chloroform or 5% FBS are summarized in Table1 with previously reported results from IC7-1 16 in chloroform for comparison. All six IC7-1 derivatives in chloroform showed a maximum excitation wavelength at 823 nm and an emission wavelength around 845 nm, both of which were similar to IC7-1 as expected. IC7-1 derivatives showed slightly higher quantum yields than the parent IC7-1. Similar results were obtained in 5% FBS.

**Table 1:** Photophysical properties of IC7-1 derivatives and IC7-1.

	Solvent	ex_max_^1^ (nm)	em_max_^2^ (nm)	Φ^3^	*ε*^4^ (mol/(L cm))
IC7-1-Me	CHCl_3_	823	841	0.07	3.3 × 10^5^
	5% FBS	823	846	0.04	1.2 × 10^5^
IC7-1-Et	CHCl_3_	823	842	0.06	3.3 × 10^5^
	5% FBS	823	847	0.04	1.3 × 10^5^
IC7-1-Pr	CHCl_3_	823	845	0.07	3.3 × 10^5^
	5% FBS	823	843	0.05	1.3 × 10^5^
IC7-1-Bu	CHCl_3_	823	845	0.06	3.3 × 10^5^
	5% FBS	823	849	0.04	1.3 × 10^5^
IC7-1-Pe	CHCl_3_	823	845	0.06	3.3 × 10^5^
	5% FBS	823	847	0.03	0.9 × 10^5^
IC7-1-He	CHCl_3_	823	845	0.06	3.3 × 10^5^
	5% FBS	823	841	0.03	0.6 × 10^5^
IC7-1 (16)	CHCl_3_	830	858	0.05	2.1 × 10^5^

1 Maximum excitation wavelength.

2 Maximum emission wavelength.

3 Quantum yield.

4 Extinction coefficient.

### Cellular localization study

**Figure 1:**
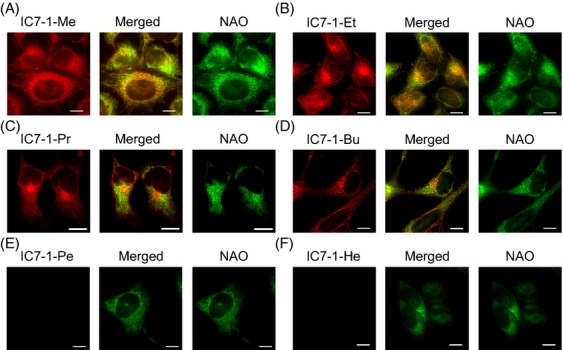
Subcellular localization of IC7-1 derivatives in HeLa cells. Cells were stained with IC7-1 derivatives (red) and NAO (mitochondria marker: green). Overlay images are shown in yellow. Scale bars are 10 *μ*m.

Representative fluorescence microscopy images of HeLa cells treated with IC7-1 derivatives and NAO (mitochondria marker dye) are shown in Figure1. Cells treated with IC7-1-Me, -Et, -Pr, and -Bu showed colocalized fluorescence with NAO indicated in white in the merged images (Fig.1A–D), while cells treated with IC7-1-Pe and -He showed weak or negligible fluorescence in the NIR region, and localization could not be discriminated in the images (Fig.1E and F).

### Cellular uptake study

**Figure 2:**
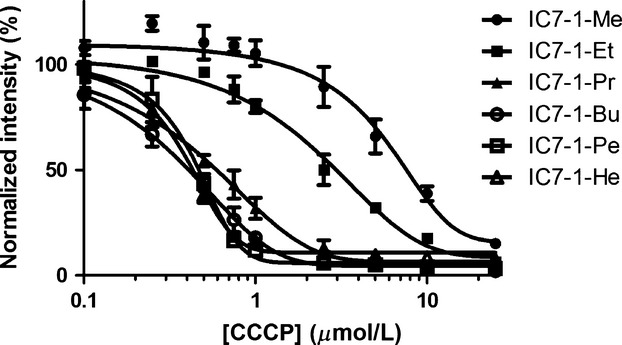
Cellular uptake of IC7-1 derivatives with an uncoupler CCCP. The cellular uptake study was performed in the presence of varying concentrations of CCCP. Data are means ± SEM of three independent experiments.

The results of cellular uptake of IC7-1 derivatives in the presence of varying concentrations of CCCP are shown in Figure2. The normalized fluorescence intensity of cells treated with IC7-1 derivatives decreased with increasing CCCP concentrations. IC7-1-Pr, -Bu, -Pe, and -He showed reduced cellular fluorescence from treatment with a low CCCP concentration compared to IC7-1-Me and -Et.

### Protein-binding assay

**Figure 3:**
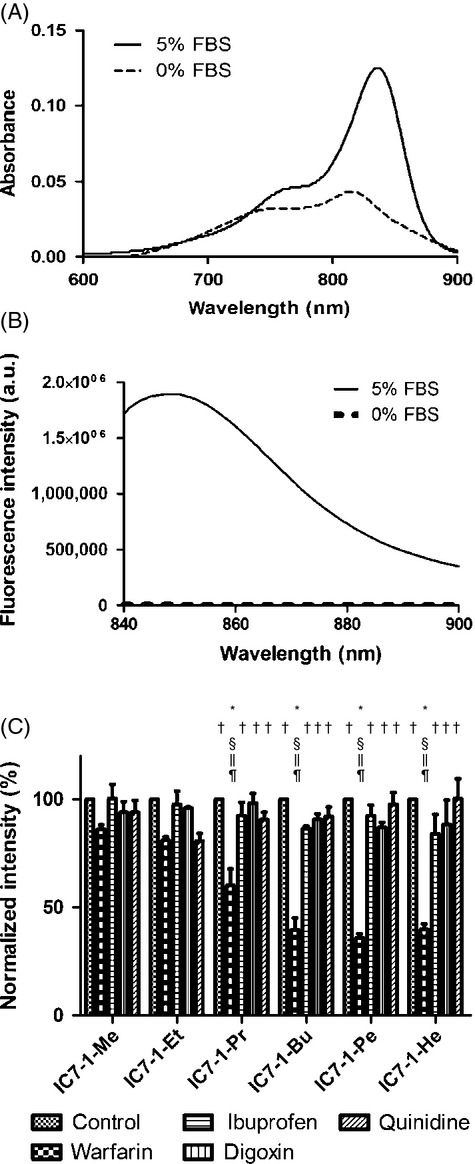
Estimation of protein binding of IC7-1 derivatives. (A) Absorbance spectra of IC7-1-Bu in 5% FBS solution or 0% FBS solution. (B) Fluorescence spectra of IC7-1-Bu in 5% FBS solution or 0% FBS solution. (C) Normalized fluorescence intensities of IC7-1 derivatives in 5% FBS solution with inhibitors of albumin or *α*_1_-glycolipoprotein. Data are means ± SD of three independent experiments. Comparisons were performed with one-way ANOVA followed by the Tukey test (*P < 0.001 vs. control, ^†^*P* < 0.001 vs. warfarin, ^§^*P* < 0.001 vs. ibuprofen, ^‖^*P* < 0.001 vs. digoxin, ^¶^*P* < 0.001 vs quinidine).

Figure3 shows (A) absorption and (B) fluorescence spectra of IC7-1-Bu in 0% and 5% FBS solutions, and (C) the normalized intensity of IC7-1 derivatives treated with inhibitors in 5% FBS solution. The absorbance peak at 836 nm and fluorescence were significantly higher in 5% FBS solution than in 0% FBS solution, which suggests that fluorescence was enhanced when IC7-1-Bu bound to proteins in FBS solution. Among the inhibitors used in the inhibitory assay (Fig.3C), warfarin considerably decreased the fluorescence of IC7-1 derivatives, in particular IC7-1-Pr to -He, in 5% FBS solution indicating that the IC7-1 derivatives bound to albumin at the warfarin-binding site. In addition, the inhibitory potency of warfarin increased in accordance with the length of the alkyl chain. Similar results were obtained in bovine serum albumin solution and mouse serum (Fig. S1).

### In vivo imaging study

**Figure 4:**
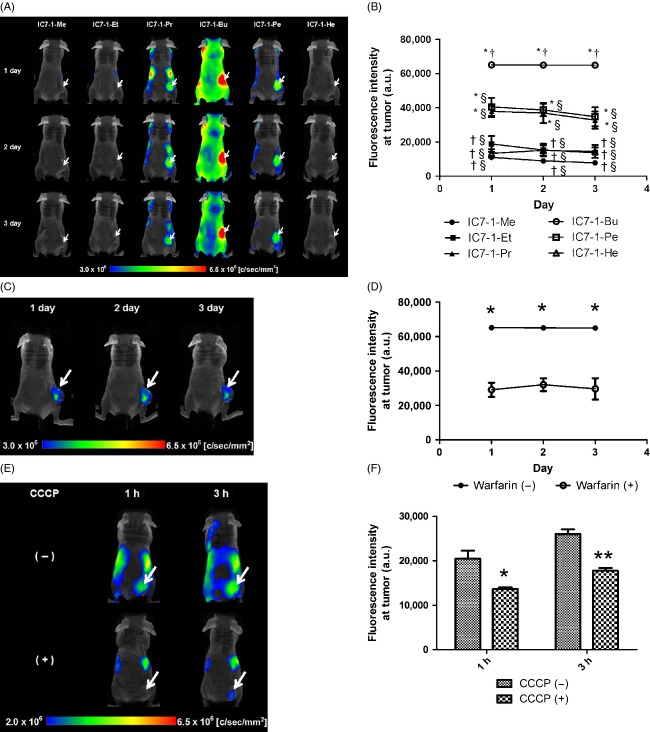
In vivo imaging of IC7-1 derivatives using HeLa cell xenograft mice. (A) Fluorescence images of HeLa cell xenograft mice 24, 48, and 72 h after intravenous administration of the IC7-1 derivative. Arrows indicate the tumor. (B) Fluorescence intensities in the tumor measured using analytical software. Data are means ± SD (*n* = 3 per group). Comparisons were performed with one-way ANOVA followed by the Tukey test (*P < 0.001 vs. IC7-1-Me, Et, or He, ^†^*P* < 0.001 vs. IC7-1-Pr or Pe, ^§^*P* < 0.001 vs. IC7-1-Bu). (C) Fluorescence images of HeLa cell xenograft mice 24, 48, and 72 h after intravenous co-administration of IC7-1-Bu and warfarin. Arrows indicate the tumor. (D) Fluorescence intensities in the tumor from (C). (E) Fluorescence images of HeLa cell xenograft mice 1 and 3 h after intravenous administration of IC7-1-Bu to CCCP pretreated mice. Arrows indicate the tumor. (F) Fluorescence intensities in the tumor from (E). Data are means ± SD (*n* = 3 per group). Comparisons were performed with one-way ANOVA followed by the Tukey test (^†^*P* < 0.001 vs. warfarin [−], *P < 0.05 vs. CCCP [−], **P < 0.01 vs. CCCP [−]).

Fluorescence images of tumor-bearing mice after administration of IC7-1 derivatives are shown in Figure4. Tumors were clearly depicted in mice treated with IC7-1-Pr, -Bu and -Pe in comparison with negligible signals from the whole body of mice treated with IC7-1-Me, -Et and -He (Fig.4A). In particular, IC7-1-Bu showed strong fluorescence intensity at the tumor region up to 72 h post administration (Fig.4B). IC7-1-Me and -Et accumulated in the liver, and were quickly excreted compared with the other derivatives (Fig. S2). The fluorescence intensity of IC7-1-He was weak at all time points.

Fluorescence images of tumor-bearing mice and region of interest (ROI) analysis data in tumors after simultaneous administration of IC7-1-Bu and warfarin are shown in Figure4C and D. Fluorescence images, obtained under the same conditions as for Figure4, exhibited the same color range. Apparently, the fluorescence intensity diminished throughout the body (Fig.4C) and led to fluorescence remaining in tumors (Fig.4D) after warfarin treatment. On the other hand, the tumor to neck fluorescence ratio did not show a significant reduction (warfarin (+) vs. warfarin (−): 2.36 ± 0.09 vs. 1.94 ± 0.11 after 1 day, 2.49 ± 0.10 vs. 2.02 ± 0.15 after 2 day, and 2.46 ± 0.09 vs. 2.07 ± 0.48 after 3 day). Fluorescence images of tumor-bearing mice successively administered CCCP and IC7-1-Bu are shown in Figure4E and F. The fluorescence intensity at the tumor was significantly decreased by CCCP pretreatment (Fig.4F).

## Discussion

In this study, our goal was to develop a NIR-DLC probe that could detect mitochondrial hyperpolarization closely related to tumorigenesis and progression 2–5 through in vivo optical imaging. Through the design, synthesis, and evaluation of series of cyanine compounds based on the core structure of the previously developed IC7-1, we found that IC7-1-Bu has great potential for this purpose. IC7-1-Bu showed fluorescence localized to the mitochondria of HeLa cells that was dependent on Δ*ψ*_m_. In addition, IC7-1-Bu enabled clear in vivo tumor imaging through serum albumin mediated transport, which has emerged as a versatile drug delivery carrier for therapeutic and diagnostic agents that diagnose and treat cancers 22–24. The results suggest that IC7-1-Bu is a promising NIR-DLC probe for in vivo tumor imaging.

Mitochondria exert both vital and lethal functions in physiological and pathological scenarios 2–5. Mitochondria are key regulators of cell death through caspase-3 activation 3,4,13 and have been implicated in multiple aspects of tumorigenesis and tumor progression through activation of oncogenes such as Ras 25,26 and Myc 27. For instance, mutations of mitochondrial or nuclear DNA that affect components of the mitochondrial respiratory chain result in inefficient ATP production, ROS overproduction, and oxidative damage to mitochondria and other macromolecules, including DNA. Furthermore, numerous polymorphisms and mutations of mitochondrial DNA correlate with increased risk of developing a variety of malignancies including breast cancer 28, prostate cancer 29, and thyroid cancer 30. Multiple hallmarks of cancer cells, including limitless proliferative potential, insensitivity to anti-growth signals, impaired apoptosis, enhanced anabolism, and decreased autophagy, have been linked to mitochondrial dysfunction 31,32, and cancer cell mitochondria are structurally and functionally different from their normal counterparts 33,34. Since hyperpolarization of mitochondria is a typical characteristic of carcinoma cells, in vivo NIR imaging of mitochondrial hyperpolarization could be a promising clinical diagnostic tool.

In fact, there have been a variety of molecular probes that target mitochondrial hyperpolarization. For instance, ^99m^Tc-MIBI, originally developed as a myocardial blood flow tracer, has been used to estimate the myocardial mitochondrial function in the clinical setting 35. Another example of such molecular probes is triphenyl phosphonium compounds that incorporate a variety of radiolabeled and fluorescent derivatives 7 and have been used as mitochondria targeting drugs 9–11,36. The development of mitochondrial hyperpolarization targeting NIR probes has been, to the best of our knowledge, less studied except for IR-780 37. Although IR-780 could clearly visualize tumors and was reported to show fluorescence in mitochondria via cell microscopy 37, an almost negligible effect of CCCP on the cellular uptake of IR-780 was observed in our cellular uptake experiment (Fig. S3), which suggested that IR-780 was probably taken up by cells independent of mitochondrial hyperpolarization. On the basis of these results, in this study, we designed and synthesized IC7-1 derivatives to optimize NIR fluorescence by focusing on the length of the alkyl side chains, which could affect the pharmacokinetics and physicochemical properties such as molecular size and lipophilicity without disturbing the fluorescence properties 38, and these compounds were investigated as NIR-DLC probes for in vivo tumor imaging.

IC7-1 is a NIR fluorescent dye that we recently developed for in vivo optical imaging (*λ*ex = 830 nm, *λ*em = 858 nm) 16. However, IC7-1 is a neutral molecule that possesses both a quaternary ammonium ion in the core cyanine moiety and a carboxyl group on the side chain. Thus, in order to fulfill the requirements of a NIR-DLC molecular probe for imaging cancer mitochondria as described above, IC7-1 required modification. We anticipated that the carboxyl group in the side chain might not be important for NIR fluorescence of IC7-1 because it is independent from the resonance network of the core cyanine moiety. Thus, in this study, the two side chains of IC7-1 were replaced with simple alkyl chains ranging from one (IC7-1-Me) to six carbons (IC7-1-He). Since an optimal range of lipophilicity for DLC probes still remained unclear 9,12 and a recent study reported that the length of the side chains of cyanine dyes greatly affected biodistribution 37, we decided to synthesize a series of IC7-1 derivatives possessing various alkyl side chain lengths and to evaluate them in in vitro and in vivo studies.

HeLa cells were used since they possess hyperpolarized mitochondria 39. To evaluate the effect of Δ*ψ*_m_ on the uptake of IC7-1 derivatives in HeLa cells, CCCP was employed as a weak acid protonophore to depolarize the cells, since it has been reported that CCCP could decrease the Δ*ψ*_m_ in a dose-dependent manner 8. As expected, the cellular uptake of IC7-1 derivatives was dependent on the Δ*ψ*_m_. In particular, the four IC7-1 derivatives possessing longer alkyl chains (IC7-1-Pr to -He) displayed higher sensitivity to membrane potential than the derivatives with shorter alkyl chains (IC7-1-Me and -Et). On the other hand, IC7-1-Me to -Bu showed brighter or at least detectable fluorescence in microscopy images of cells compared with IC7-1-Pe and -He. The results of these cell experiments indicated that IC7-1-Pr and -Bu had the best potential for in vivo imaging and were subjected to further evaluation.

As for transport of IC7-1 derivatives across cell membranes, it was curious that IC7-1 derivatives with longer alkyl chains, that is, more lipophilic compounds, were less taken up by cells, which implied the involvement of a cell membrane transport system besides passive diffusion. We evaluated the involvement of anion transporters on the uptake of IC7-1 derivatives in a similar method as previously reported for the cationic cyanine dye IR-780 37 and found that transporters were involved in the cellular uptake of IC7-1 derivatives to a small degree (Fig. S4). In addition, since IC7-1 derivatives tend to form H aggregates in aqueous solutions without proteins, as identified in absorbance spectra, similar to cyanine dyes with long alkyl chains in their structures 40,41, there might be a lower free fraction of IC7-1 derivatives possessing longer alkyl chains in additive buffer that could passively diffuse into cells, compared to IC7-1 derivatives possessing shorter alkyl chains. Although a precise transport mechanism for IC7-1 derivatives remains unclear, passive diffusion and transporters might provide cooperative effects. In any case, the results suggest that replacement of the side chains of IC7-1 with alkyl chains could be effective for the development of a NIR-DLC probe that detects the change in Δ*ψ*_m_ in cancer cells.

Results from the biodistribution study showed that the IC7-1 derivatives exhibited significantly different distribution behavior. From the ROI analysis of tumors, the IC7-1 derivatives were classified into three groups as follows: (1) IC7-1-Bu in the high intensity group, (2) IC7-1-Pr and -Pe in the mild intensity group, and (3) IC7-1-Me, -Et, and -He in the negligible intensity group. In particular, IC7-1-Bu clearly delineated tumors, and its fluorescence was retained for 72 h after i.v. administration compared to the other derivatives. Such long-lasting retention in tumors is a desirable characteristic of DLCs 39,42,43. The microscopy results showing a higher cellular uptake for IC7-1 derivatives possessing shorter alkyl chains appeared to be inconsistent with in vivo results in which the highest fluorescence intensity was found for IC7-1 derivatives possessing mid-length alkyl chains (groups 1 and 2). Therefore, we focused on the involvement of serum proteins as a drug delivery carrier 22–24 for tumor targeting of IC7-1 derivatives and performed an in vitro blocking study using albumin-binding inhibitors. In the case of indocyanine green (ICG), initial fluorescence emission of i.v. administered ICG after binding to albumin in the blood stream was followed by rapid clearance through the liver into feces with low accumulation in tumors 44. All of the IC7-1 derivatives emitted fluorescence in solutions that included proteins regardless of specific binding (Figs.3A, B, and S1), and in particular, IC7-1 derivatives with longer alkyl chains (IC7-1-Pr to -He) specifically bound to albumin at the warfarin-binding site (albumin site I 20,45,46, Fig.3C). In addition, moderate binding affinity between the drug and albumin was reported to be essential for the use of albumin as a tumor targeting carrier that targets tumors based on the enhanced permeability and retention (EPR) effect followed by release of the drug from albumin and uptake inside tumor cells 22–24. In fact, IC7-1-Pr, -Bu, and -Pe, which specifically bound to albumin (Figs.3C and S1) with moderate to high-binding constants (Table S1) among IC7-1 derivatives, depicted tumors. Moreover, in an in vivo blocking study, the tumor to neck fluorescence ratio did not change with warfarin treatment. These results indicate that the fluorescence intensity over a wide region of the whole body was due to IC7-1-Bu bound to albumin and that IC7-1-Bu not bound to albumin was not delivered to the tumor region. Therefore, the correct balance between albumin-binding affinity and cellular uptake efficiency could provide a rationale for the in vivo results of IC7-1-Bu. The fact that the tumor fluorescence of IC7-1-Bu was modulated by CCCP pretreatment strengthens the importance of the balance. The albumin-binding affinity of IC7-1 derivatives would also account for low accumulation in mitochondria-rich organs such as heart and kidney 7. Nevertheless, it should be emphasized that IC7-1-Bu was the most promising probe among the six compounds evaluated in this study.

As for limitations of probes targeting mitochondrial hyperpolarization, precise evaluation of probe utility in ex vivo and/or in vivo experiments remains unclear because Δ*ψ*_m_ is not sustained in prepared tissue sections 47,48. In fact, other DLC probes such as triphenyl phosphonium compounds have been evaluated in in vitro experiments using cells and depolarization agents 8,49 similar to the protocol in our study. Therefore, the utility of IC7-1-Bu should be compared with established DLC probes in ex vivo and/or in vivo experiments. While anticancer agents such as apoptosis-inducing ligands could be used to evaluate probe utility in vivo, these agents could exert functional changes on tumors but would not directly affect the mitochondrial potential. Perhaps more simply, an in vivo imaging study using a large number of mice bearing tumors derived from a variety of tumor cells possessing diverse mitochondrial potential, as measured in in vitro experiments, should be performed to analyze the relationship between tumor imaging quality and cellular mitochondrial potential. In addition, cytoplasmic hybrid (cybrid) technology, which can exchange the mitochondria of two different cells, could contribute to the interpretation of Δ*ψ*_m_ in vivo 50.

In conclusion, we have synthesized and evaluated a series of cationic cyanine dyes with varying alkyl chain lengths that provided a wide range of lipophilicities and serum albumin-binding rates. IC7-1-Bu showed fluorescence localized to the mitochondria of HeLa cells that was dependent on Δ*ψ*_m_ and also enabled clear in vivo tumor imaging via serum albumin as a drug carrier for effective tumor targeting. These findings suggest that IC7-1-Bu is a promising NIR probe for in vivo tumor imaging.

## Supporting Information

**Figure S1**. Protein binding assay of IC7-1 derivatives in bovine serum albumin solution and mouse serum. **Figure S2.** In vivo imaging study of IC7-1 derivatives. **Figure S3.** Comparison of cellular uptake of IC7-1-Bu and IR-780 under CCCP treatment. **Figure S4.** Estimation of cellular uptake of IC7-1-derivatives via an organic anion transporter.

**Table S1.** Binding constants of IC7-1 derivatives

**Data S1.** Supplemental method.
