# Supplementary material for: Investigation of cyanine dyes for in vivo optical imaging of altered mitochondrial membrane potential in tumors
Source: Cancer Med. 2014 Apr 16;3(4):775–86. doi: 10.1002/cam4.252 (PMC4303146; doi:10.1002/cam4.252)
Supplement: Supplementary file 1 [file cam40003-0775-sd1.ppt]

## Slide 1
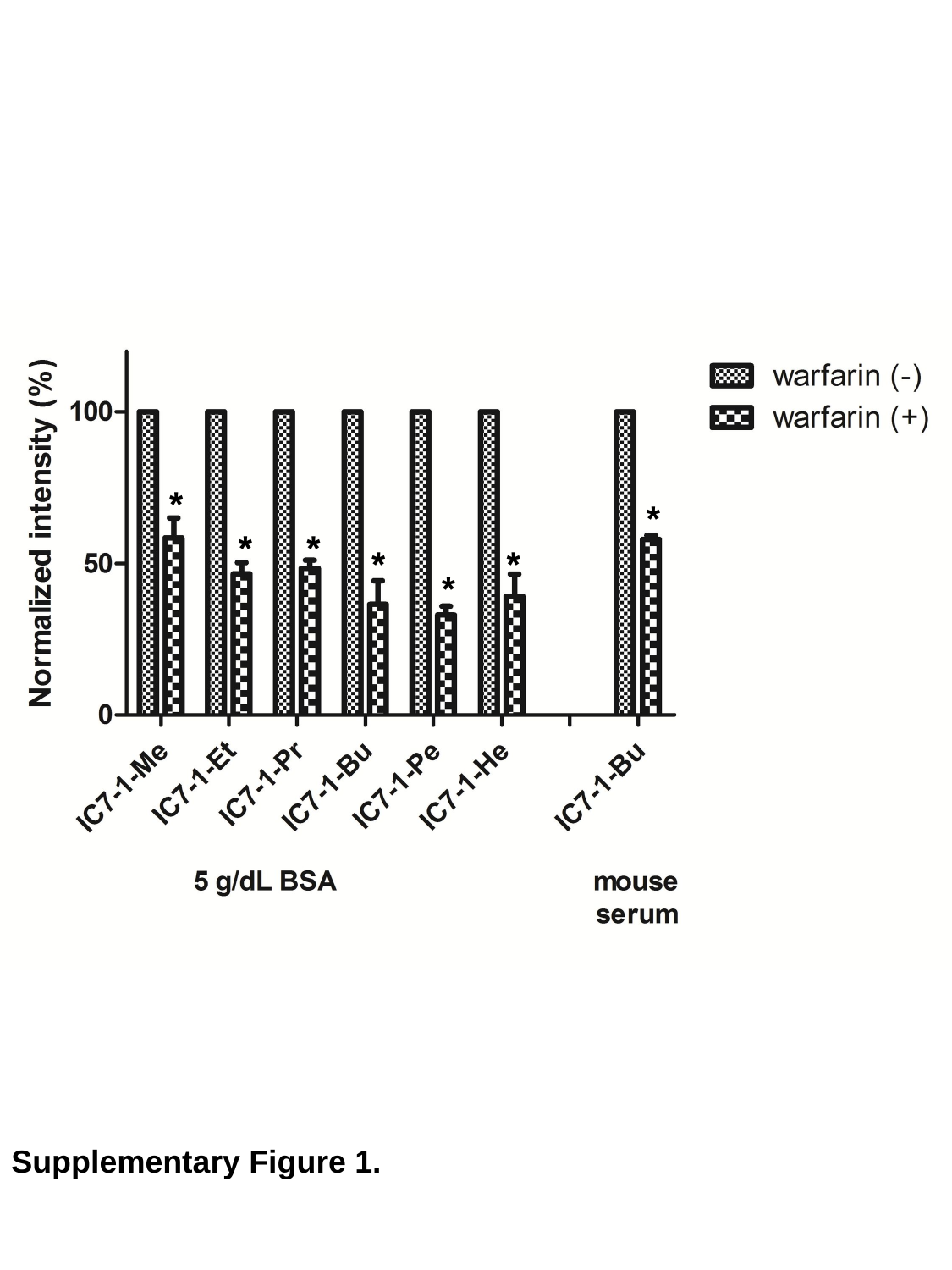

*
*
*
*
*
*
*
Supplementary Figure 1.

## Slide 2
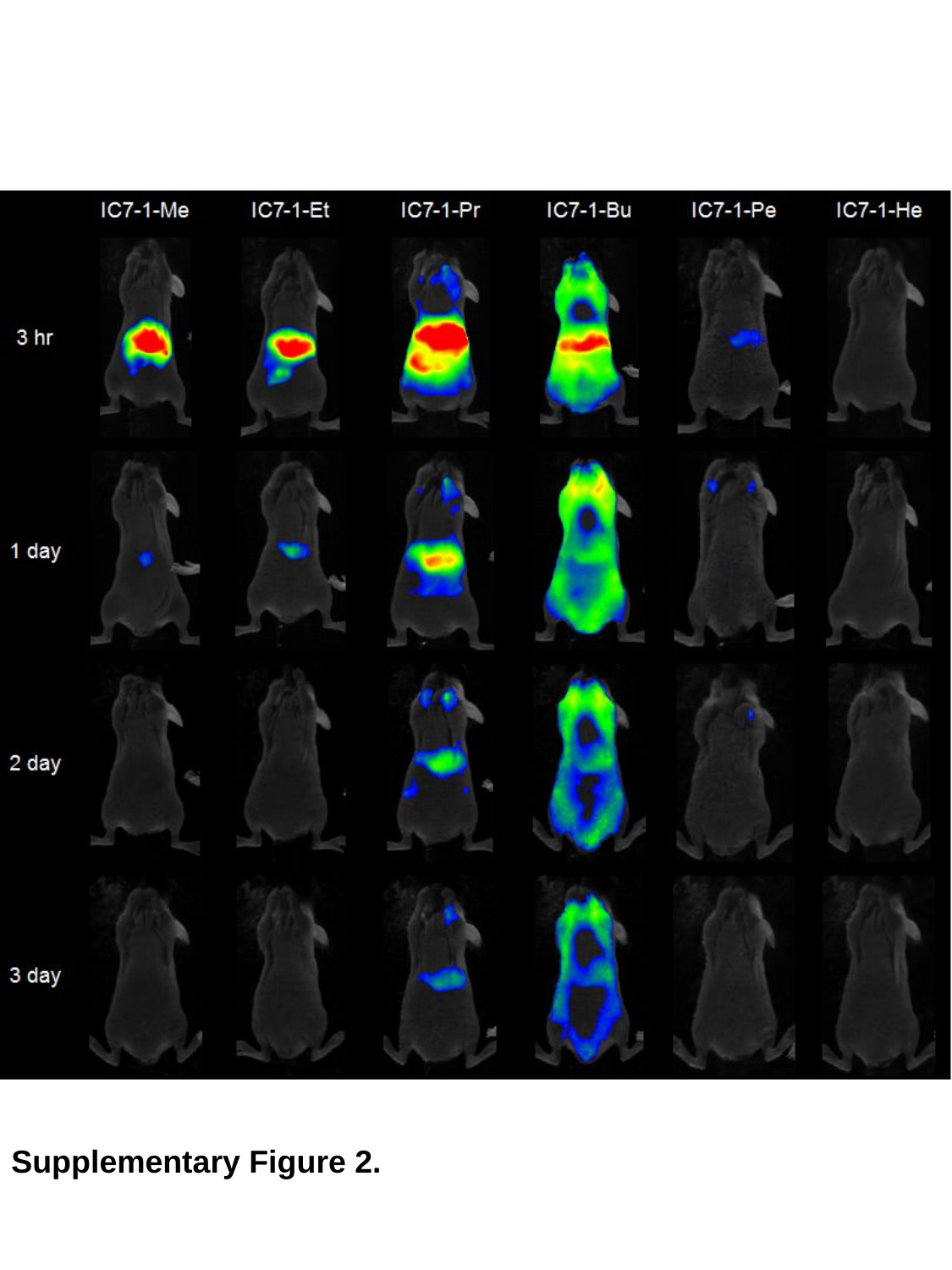

Supplementary Figure 2.

## Slide 3
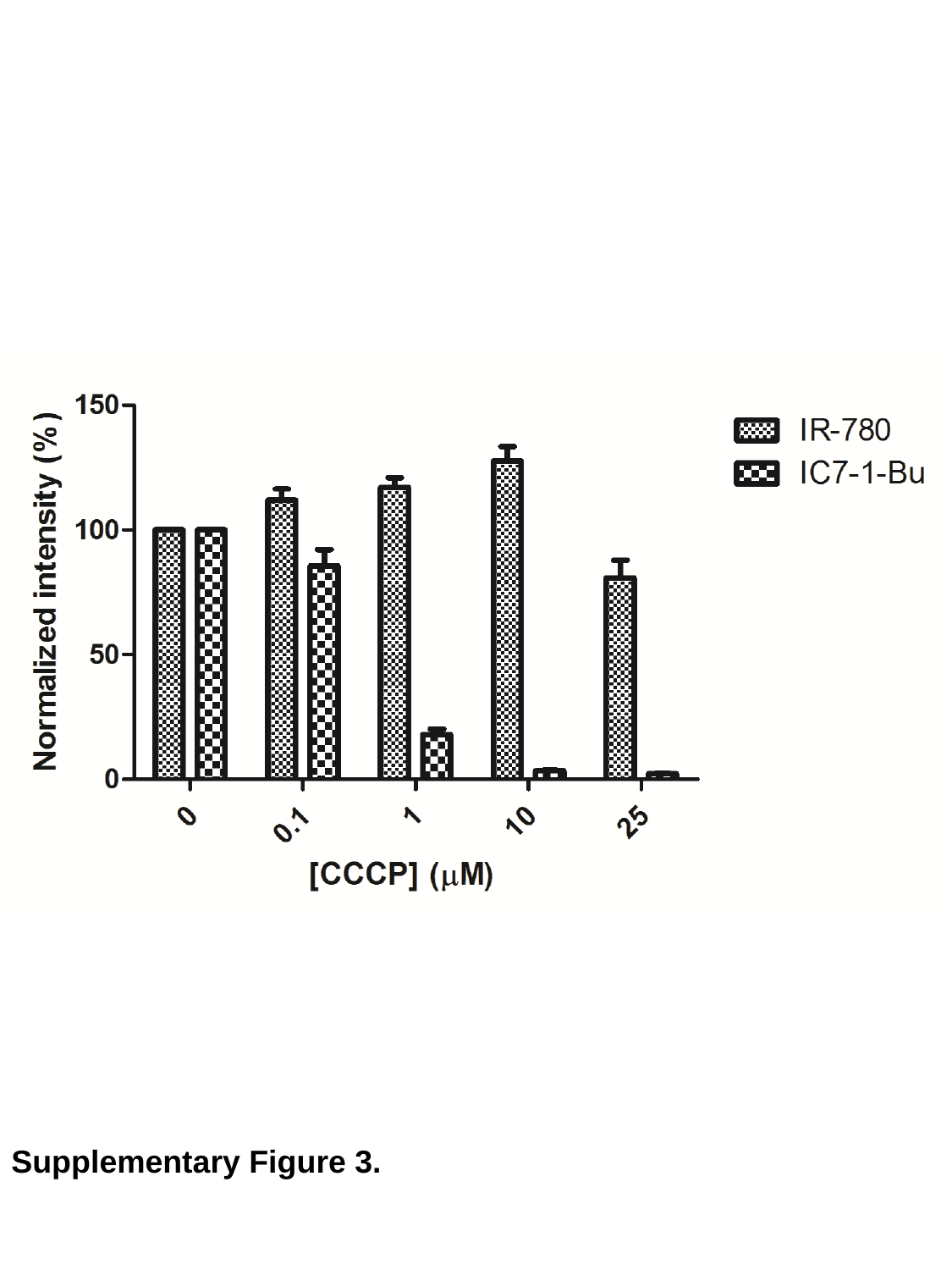

Supplementary Figure 3.

## Slide 4
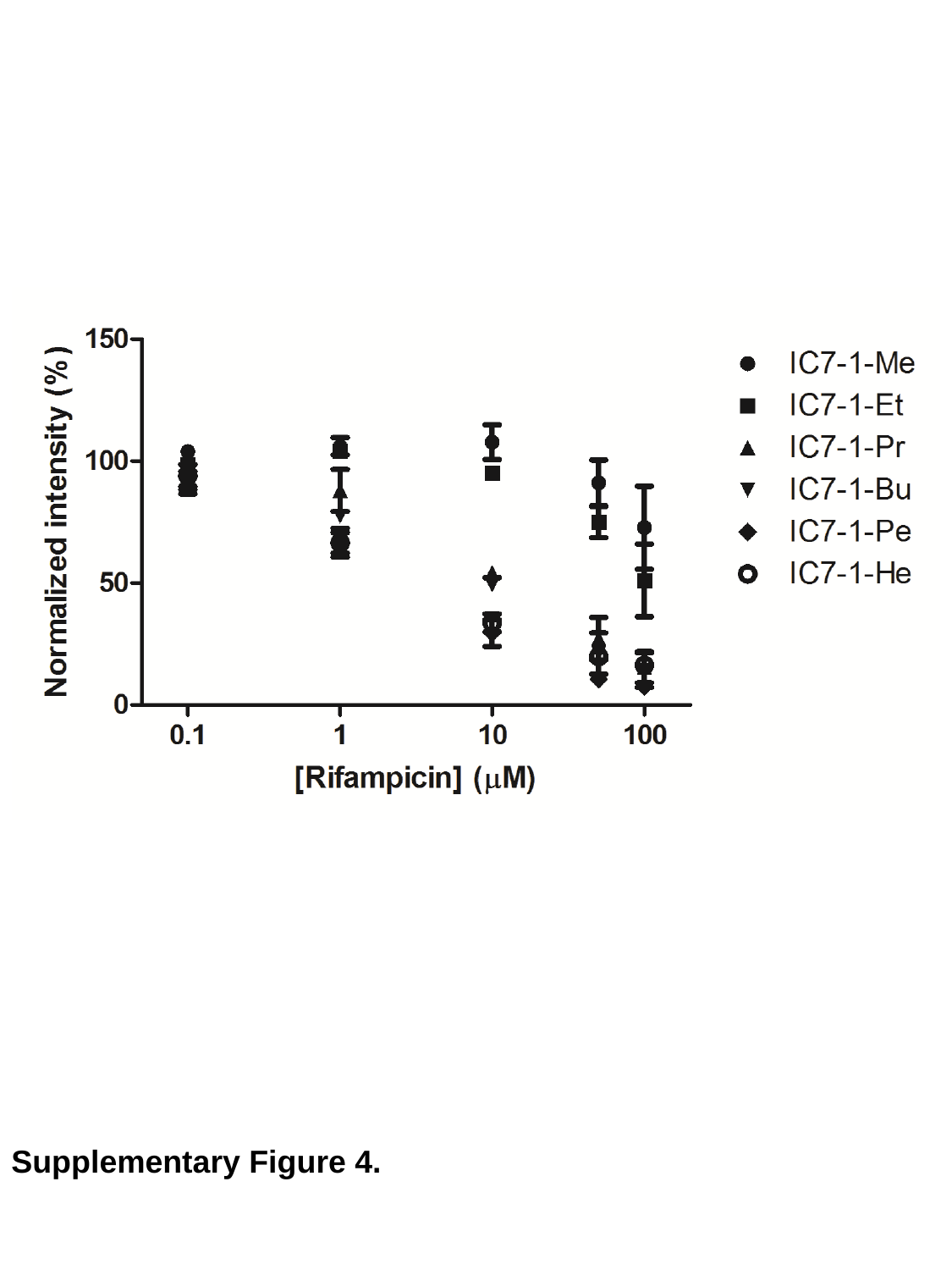

Supplementary Figure 4.
