# Supplementary material for: Investigation of cyanine dyes for in vivo optical imaging of altered mitochondrial membrane potential in tumors
Source: Cancer Med. 2014 Apr 16;3(4):775–86. doi: 10.1002/cam4.252 (PMC4303146; doi:10.1002/cam4.252)
Supplement: Supplementary file 2 [file cam40003-0775-sd2.doc]

**Supplementary Table 1: Binding constants of IC7-1 derivatives**

|  | IC7-1-Me | IC7-1-Et | IC7-1-Pr | IC7-1-Bu | IC7-1-Pe | IC7-1-He |
| --- | --- | --- | --- | --- | --- | --- |
| Ka (x 10^6^) | 1.3 | 1.8 | 2.4 | 2.1 | 1.6 | 1.2 |
