# Supplementary material for: Investigation of cyanine dyes for in vivo optical imaging of altered mitochondrial membrane potential in tumors
Source: Cancer Med. 2014 Apr 16;3(4):775–86. doi: 10.1002/cam4.252 (PMC4303146; doi:10.1002/cam4.252)
Supplement: Supplementary file 3 [file cam40003-0775-sd3.doc]

**Supplemental method**

**Association constants of IC7-1 derivatives to albumin (Supplementary Table 1)**

Binding studies of IC7-1 derivatives to albumin were conducted as reported previously (Beckford G *et al*. The solvatochromic effects of side chain substitution on the binding interaction of novel tricarbocyanine dyes with human serum albumin. *Talanta* 2012;92: 45-52). Briefly, IC7-1 derivatives (1 μM final concentration) were incubated with BSA (0, 0.4, 0.6, 0.8, and 1.0 μM) for 30 min to equilibrate. The fluorescence intensity was measured following the excitation at 823 nm and the emission from 840 to 900 nm. The interaction between dye D and albumin A can be expressed by Eq. 1:

D + A ↔ DA (1)

The association constant for the dye-albumin complex formation can be expressed by Eq. 2:

K_a_ = [DA]/([D][A]) (2)

Then, the association constant K_a_ of the dye-albumin complex was calculated from the data for the fluorimetric titration of the dye with albumin using Eq. 3:

1/ΔF = 1/k[D] + (1/(k[D]K_a_))·(1/[A]) (3)

where k is a constant depending on the instrumentation and quantum efficiency of the process.

Therefore, a plot of the reciprocal of ΔF versus the reciprocal of albumin concentration gives a straight line. The association constant was calculated by dividing the intercept by the slope of the line.

**Protein binding assay in bovine serum albumin solution (Supplementary Figure 1)**

Warfarin (0 and 3.79 mM), as a competitive inhibitor of albumin binding site I, was dissolved in bovine serum albumin (BSA) solution in PBS (5 g/dL) or mouse serum obtained from 8 week old female BALB/c mice, and the mixture was incubated for 30 min at r.t. Immediately after the addition of IC7-1 derivatives (1 μM at final concentration), the fluorescence intensity was measured following the excitation at 823 nm and the emission from 840 to 900 nm. The normalized intensity (%) was calculated as a percentage of fluorescence intensity obtained from an inhibitor untreated sample.

***In vivo* imaging study (Supplementary Figure 2)**

The mice were the same as were used in the *in vivo* imaging study (Figure 4 (A)). The mice were imaged from the ventral side.

3, 24, 48 and 72 h after intravenous administration of the IC7-1 derivative (10 nmol, 100 μL), tumor-bearing mice under anesthesia with 2.5% isoflurane gas in oxygen flow (1.5 L/min) were imaged by Clairvivo® OPT (SHIMADZU Co., Kyoto, Japan) with a 785 nm single laser for excitation and a 845/55 nm band-path filter for emission. Exposure time was 5s for the scan at 3 h, and 3s for the other scans. Clairvivo® OPT measurement and display software ver. 2.6.0.0. (SHIMADZU Co., Kyoto, Japan) was used for imaging analysis.

**Comparison of cellular uptake of IC7-1-Bu and IR-780 under CCCP treatment (Supplementary Figure 3)**

The buffer solution (pH 7.4) for the cellular uptake study was composed of NaCl (145 mM), KCl (5.4 mM), CaCl_2_ (1.2 mM), MgSO_4_ (0.8 mM), NaH_2_PO_4_ (0.8 mM), dextrose (5.6 mM), and HEPES (5 mM). The HeLa cells (1 x 10^6^ cells / ml buffer solution) were incubated with an uncoupler (CCCP; 0, 0.1, 0.25, 0.5, 0.75, 1, 2.5, 5, 10, and 25 μM) for 5 min at 37 ºC in 1.5 mL microtubes, followed by the addition of IR-780 or IC7-1-Bu (1 μM final concentration) After a 60 min incubation with the dyes, the cells were washed twice and resuspended with buffer solution. Fluorescence intensity was measured following the excitation at 763 nm and the emission from 780 to 880 nm for IR-780, and the excitation at 823 nm and the emission from 840 to 900 nm for IC7-1-Bu. The normalized intensity (%) was calculated as a percentage of fluorescence intensity obtained with CCCP untreated (0 μM) cells.

**Estimation of cellular uptake of IC7-1-derivatives via an organic anion transporter (Supplementary Figure 4)**

The HeLa cells (1 x 10^6^ cells / ml buffer solution) were incubated with an organic anion transporter (OATP) inhibitor (rifampicin; 0, 10, 25, 50, and 100 μM) for 10 min at 37 ºC in 1.5 mL microtubes, followed by the addition of the IC7-1 derivative (1 μM final concentration). After a 60 min incubation with the dyes, the cells were washed twice and resuspended with buffer solution. Fluorescence intensity was measured following the excitation at 823 nm and the emission from 840 to 900 nm. The normalized intensity (%) was calculated as a percentage of fluorescence intensity obtained with rifampicin untreated (0 μM) cells.

**Statistical analysis**

In the cellular uptake study, data are expressed as means ± SEM. Otherwise, data are expressed as means ± SD. Data were analyzed with one-way factorial ANOVA followed by the Tukey test, and values with *p* < 0.05 were considered significant.
